# Controlling antimicrobial resistance: Interfering in the process of natural selection

**DOI:** 10.1186/2047-2994-2-32

**Published:** 2013-11-18

**Authors:** Johan W Mouton

**Affiliations:** 1 Department of Medical Microbiology, Radboud University Nijmegen, Geert Grooteplein-Zuid 10, Route 777, 6525 GA Nijmegen, The Netherlands

## 

In this issue of *Antimicrobial Resistance and Infection Control* Huttner and colleagues portray a global view from the World Healthcare-Associated Infections Forum –the fourth in the series - on a familiar topic: the rapid spread of antimicrobial resistance [1]. The terminology ‘familiar’ is used here with some emphasis. The increasing number of papers appearing every year that cry out against the increasing and rapid emergence of resistance and a future without antibiotics seem to go hand in hand with, to speak in statistical terms, a correlation coefficient that approaches 1. There are, however, a number of issues that need well deserved attention and it is in the referred position paper that these are stated together in a comprehensive review. With an effort not to repeat everything said already, there are a few of these issues that need and warrant highlighting, and these are not so much the problems that are faced, but more the potential solutions towards it. And then it all comes back to interfering with the two basic principles that govern emergence and spread of resistance. The first is natural selection (a term used to describe the process *in sensu stricto*, although admittedly the very fact that resistance will emerge under selection pressure does have a teleological ring to it) and the second dissemination – interfering in the latter being a specific element of the title of the Journal.

Natural selection will, as a basic process in nature, always give rise to emergence of resistance to antimicrobials in micro-organisms in the presence of antimicrobials, be it that the frequency (or probability of occurrence) is of course dependent on many factors. Although the continuous cry for new antimicrobials is certainly and highly justified, and mechanisms to stimulate development of new antimicrobials may even require more attention than presently is the case, these new agents will be losing their potential as soon as they become available for use if no mechanisms are put in place to restrict its usage in a sensible manner. As noted by Huttner and colleagues, the present situation was prophesied by Alexander Fleming in his acceptance speech of the Nobel prize in 1945. Yet, in the ensuing years, there was a sales and marketing process of almost any drug that could be found that had some antimicrobial activity and indications to use became manifold, often without too much evidence. Serious problems with other drugs put an end to that process through the Kefauver and Harris Amendments in 1962 –at least in the US– although it would take years before rigorous testing and development became the standard. But use still appears to be manifold – how else can it be explained that the per capita use of antimicrobials is almost 20 fold more in one country than that in another?

The main challenge therefore is how to influence the process of natural selection, in other words how to minimize exposure of micro-organisms to antimicrobials without compromising their efficacy or restricting its use to those patients that need them. Or from a different perspective, optimizing the use of antimicrobials that we have and thereby preserving antibiotics for the future. One of the obvious answers is formulated by Huttner and colleagues: antibiotic conservation or stewardship programs. These programs are increasingly receiving attention as more and more evidence is collected suggesting that these actually work. As an example, The Netherlands government recently issued a statement strongly supporting the installation of so-called A-teams (antibiotic stewardship teams) in every hospital in country with the Health Inspectorate given the assignment to oversee that, within two years, these have come true. The A-teams are typically multidisciplinary and will consist of a clinical microbiologist, an infectious disease physician and a hospital pharmacist. Yet even these teams cannot function without proper guidance of evidence based criteria. What is the optimal duration of therapy? What is the optimal dosing regimen and antimicrobial choice? These are all questions that clearly need to be answered and will require a huge effort.

It is obvious that antimicrobial stewardship and hospital hygiene measures introduce costs that hospital directors are not happy to be confronted with in the face of constrained budgets. This is even more the case in long-term care facilities and nursing homes. Yet in the long run, prevention of resistance is not only for the benefit for patients, it is less expensive as well. With the (probably under-) estimated cost of 55 billion per year in the US alone, any measure that significantly reduces resistance will be cost-effective for society as a whole. This requires mechanisms in society that promote these measures. Unfortunately, these are still lacking both in the developed world as well as in third world countries. There is clearly something wrong in the checks and balances of the system.

As the authors indicate, it is not only human consumption that requires measures, but also the extensive use in animal husbandry. Using The Netherlands again as an example, it is noteworthy that antimicrobial use in humans in The Netherlands has traditionally been one of the lowest in Europe, but at the same time one of the highest in animal husbandry. This paradox was recognized by the government a few years ago and targets to reduce antibiotic consumption and thereby exposure were set. To reach these goals, other stringent measures were taken over time: farmers now have to report antimicrobial use in a central database and, on the prescription side, veterinarians have to report as well. Taken together, a significant reduction in sales and use has been reached in just a couple of years.

These examples demonstrate that if certain check and balance mechanisms are imposed, interfering in the process of natural selection is possible. However, it is not an easy process as any change in behaviour is not easy and certainly not always accepted. This is clearly demonstrated by the opposition and demonstrations by French veterinarians this week against an upcoming law supposedly restricting the right to prescribe certain antibiotics (which in some other countries has been put into law for some time already!).

The continuing use, or rather abuse of antibiotics favours emergence and spread of resistance. Micro-organisms are not restricted by borders, do not care whether they are in the hospital or outside. They thrive wherever they have an opportunity to do so and we, as humans, have created that environment for resistant bacteria. This needs to stop. The concluding remarks in the Huttner *et al*., [1] paper are more than justified, but can be summarized as: action now, now, now! It might not be too late.

## Competing interest

The author declares that he has no competing interest.
